# Diagnostic value of peripheral blood leukocyte parameters, NE-WX, LY-Y, MO-WY, MO-Y, MO-Z, and NE-SFL, in lung cancer

**DOI:** 10.3389/fonc.2026.1793608

**Published:** 2026-08-26

**Authors:** Wenqian Tian, Youhua Yuan, Yahui Hu, Lu Bai, Lan Gao

**Affiliations:** 1 Department of Special Laboratory, Henan Provincial People’s Hospital, People’s Hospital of Zhengzhou University, People’s Hospital of Henan University, Zhengzhou, Henan, China; 2 Medical Clinical Laboratory, The Fifth Clinical Medical College of Henan University of Chinese Medicine/Zheng Zhou People’s Hospital, Zhengzhou, Henan, China; 3 Medical Records Office, Henan Provincial People’s Hospital, People’s Hospital of Zhengzhou University, People’s Hospital of Henan University, Zhengzhou, Henan, China

**Keywords:** biomarkers, cell population data, diagnostic value, lung cancer, machine learning

## Abstract

**Introduction:**

We aimed to assess the diagnostic potential of six leukocyte parameters (NE-WX, LY-Y, MO-WY, MO-Y, MO-Z, and NE-SFL) for lung cancer by analysing 423 patients, 39 benign cases, and 346 healthy controls.

**Methods:**

The results of these six leukocyte parameters were compared among groups using the Kruskal–Wallis H test. Multivariate logistic regression was used to analyze correlations between NE-WX, LY-Y, MO-WY, MO-Y, MO-Z, NE-SFL, and lung cancer diagnosis. Patients were randomly grouped into training and testing sets, and nine machine-learning models (support vector machine, gradient boosting machine, artificial neural network, random forest [RF], extreme gradient boosting, K-nearest neighbor, adaptive boosting, light gradient boosting machine, and catboost) were built to compare the diagnostic performances. The DeLong test was adopted to compare the area under the receiver operating characteristic curve (AUC) values among nine machine learning models.

**Results:**

These six leukocyte parameter levels differed significantly between the lung cancer and control groups. Multivariate logistic regression revealed a strong predictive value of NE-WX, LY-Y, MO-WY, MO-Y, MO-Z, and NE-SFL for lung cancer. NE-WX and NE-SFL were independent risk predictors. Receiver operating characteristic analysis revealed that MO-Y was the best diagnostic single indicator for lung cancer (AUC = 0.787, specificity=82.0%). The combination of LY-Y, MO-Y, MO-Z, and NE-SFL demonstrated superior diagnostic efficacy (AUC = 0.897, sensitivity=70.6%, specificity=78.9%, Youden index=0.495) compared with any single indicator. The RF model exhibited high consistency between predicted and actual values, demonstrating robust and reliable predictive performance in lung cancer diagnosis. MO-Y and MO-Z were the top predictive biomarkers with the strongest predictive value.

**Conclusions:**

NE-WX, LY-Y, MO-WY, MO-Y, MO-Z, and NE-SFL may aid in diagnosis of lung cancer. The RF model demonstrates strong potential for lung cancer detection.

## 1 Introduction

Lung cancer (LC) remains a leading cause of cancer-related morbidity and mortality worldwide and represents a significant public health challenge (1, 2). Although targeted therapy and immunotherapy have improved treatment outcomes, the 5-year survival rate remains low. By 2050, LC is projected to become the leading cause of cancer-related deaths worldwide (3). Within the epidemiological landscape of China, LC is the predominant contributor to both cancer incidence and mortality (4). LC is traditionally classified into non-small cell LC (NSCLC) and small cell LC (SCLC) based on the histological type. NSCLC is the predominant histological subtype, responsible for an estimated 80–85% of the total case burden. NSCLC includes adenocarcinoma and squamous cell carcinoma, whereas SCLCs account for the remaining subtypes. The clinical manifestations of the different pathological types of LC are largely similar. Most patients are asymptomatic early and present at mid-to-advanced stages, resulting in missed opportunities for curative treatment. Hence, early detection and diagnosis of LC are paramount for improving survival rates and reducing mortality (5, 6).

Histopathological examination remains the gold standard for the diagnosis of LC. However, due to its invasive nature, technical difficulty, and variable diagnostic yields across biopsy techniques, its clinical applicability is limited. Serum tumor markers can aid in the diagnosis of LC and in the differential diagnosis of pathological subtypes. Commonly used detection indicators include carcinoembryonic antigen (CEA), cytokeratin 19 fragment antigen 211 (CYF211), neuron-specific enolase (NSE), and squamous cell carcinoma antigen (SCCA). However, when these indicators are used alone, their differential diagnostic efficacy for LC remains poor. Therefore, combined marker assessment is required to improve sensitivity and specificity in clinical applications (7). Currently, an ideal screening or early diagnostic model for LC is lacking.

The Sysmex XN series is a fully automated blood analyzer that uses fluorescence flow cytometry to measure blood cells (5). In addition to complete blood count parameters, various cell population data (CPD) reflecting leukocyte activation/maturation states can also be generated (8). These include absolute count of immature granulocytes (IG), absolute concentration of high-fluorescence large cells (HFLC), neutrophil forward scattered light distribution width, neutrophil lateral scattered light distribution width (NE-WX), neutrophil lateral fluorescence distribution width, lymphocyte forward scattered light distribution width, lymphocyte lateral scattered light distribution width, lymphocyte lateral fluorescence intensity distribution width, monocyte lateral fluorescence distribution width (MO-WY), monocyte lateral scattered light distribution width, monocyte forward scattered light distribution width, lymphocyte lateral scattered light intensity, lymphocyte fluorescence intensity (LY-Y), lymphocyte forward scattered light intensity, monocyte lateral scattered light intensity, monocyte fluorescence intensity (MO-Y), monocyte forward scattered light intensity (MO-Z), neutrophil lateral scattered light intensity, neutrophil fluorescence intensity (NE-SFL), and neutrophil forward scattered light intensity. These leukocyte parameter sets are designed to measure inherent biological properties, namely complexity, nucleic acid content, and size, that distinguish individual cell types during analytical differentiation. These CPD parameters have shown utility in conditions, such as sepsis and leukemia (6, 8, 9). Lin et al. indicated that the percentage changes in HFLC and IG in patients with acute pancreatitis during hospitalization can predict the development and prognosis of the disease (10–12). In patients with COVID-19 complicated by cytokine storms, CPD parameters underwent significant changes (13). An expanding body of evidence links CPD parameters to a growing range of diseases. In this study, we retrospectively collected peripheral blood leukocyte CPD parameters, and their distribution was analysed to identify new biomarkers for early diagnosis and treatment monitoring of LC. Simultaneously, nine machine-learning models were constructed, and the model with the best diagnostic performance was selected as the most effective tool for LC diagnosis.

## 2 Materials and methods

### 2.1 Clinical characteristics

The study group enrolled 423 patients with newly diagnosed LC, as confirmed by clinical manifestations and pathological examination according to the 2021 World Health Organisation histological classification criteria for lung tumors (14). They were diagnosed and treated at Henan Provincial People’s Hospital from July 2022 to September 2023 and included 227 men and 196 women, with a median age of 60 (range: 20–86) years. Moreover, the study included 39 patients who had benign lung tumors. Definitive diagnosis of benign lung tumors was based on pathological findings from the surgically excised specimens. There were 8 men and 31 women, aged 21–80 (median: 54) years. No patient had severe infections, a history of tumors in other organs, or diseases of the heart, liver, or kidneys; patients with autoimmune and hematological system diseases were excluded. No patient received hormone therapy, radiotherapy, or chemotherapy before surgery. For the control group, 346 healthy individuals were enrolled from the Physical Examination Centre of Henan Provincial People’s Hospital over the same time frame. The group comprised 201 men and 145 women, with a median age of 59 (range: 54–64) years. The inclusion criteria for healthy individuals were normal liver and kidney function, absence of inflammation or other underlying diseases, and no history of systemic malignancy or inflammatory disease. Individuals with a recent history of a cold, fever, trauma, or surgery within 1 month before blood collection were excluded based on a review of their medical records. This study was approved by the Medical Ethics Committee of Henan Provincial People’s Hospital (Approval No. 567). All patient data used in this study were fully de-identified to protect personal privacy.

### 2.2 Test protocol

Peripheral blood samples were collected from the healthy control group on the day of the physical examination, and from patients with LC and patients with benign lung tumors after admission. Blood samples were obtained in tubes containing EDTA-K_2_ anticoagulant and analyzed using a Sysmex XN-2000 blood analyzer within 4 h of collection, following the standard operating procedures of the laboratory. The following white blood cell parameters of each group were collected: white blood cell (WBC) count, absolute neutrophil value (NEU), absolute lymphocyte value (LYM), absolute monocyte count (MONO), PLT, NE-WX, LY-Y. MO-WY MO-Y, MO-Z, and NE-SFL. Information, such as sex, age, smoking history, drinking history, and laboratory test data, including CEA, CYF211, NSE, and SCCA, was obtained from the electronic medical record system.

### 2.3 Statistical analysis

Statistical analysis was conducted using IBM SPSS Statistics for Windows, version 26.0 (IBM Corp., Armonk, NY, USA), and figures were created using Prism software (version 8.0; GraphPad Software, San Diego, CA, USA). Depending on their distribution, non-normally distributed variables are presented as median (P25, P75). According to the data distribution, one-way analysis of variance or the Kruskal–Wallis H test was used to compare differences among the LC, benign tumor, and healthy control groups, followed by Bonferroni correction for multiple comparisons. Multivariable logistic regression analysis was performed using the LC status as the dependent variable to evaluate the association between peripheral blood WBC parameters and the occurrence of LC. A receiver operating characteristic (ROC) curve was plotted, and the area under the ROC curve (AUC), sensitivity, and specificity were calculated to evaluate the diagnostic efficacy of WBC parameters in predicting LC. A *p*-value <0.05 was considered statistically significant.

### 2.4 Machine-learning models

**Figure 1:**
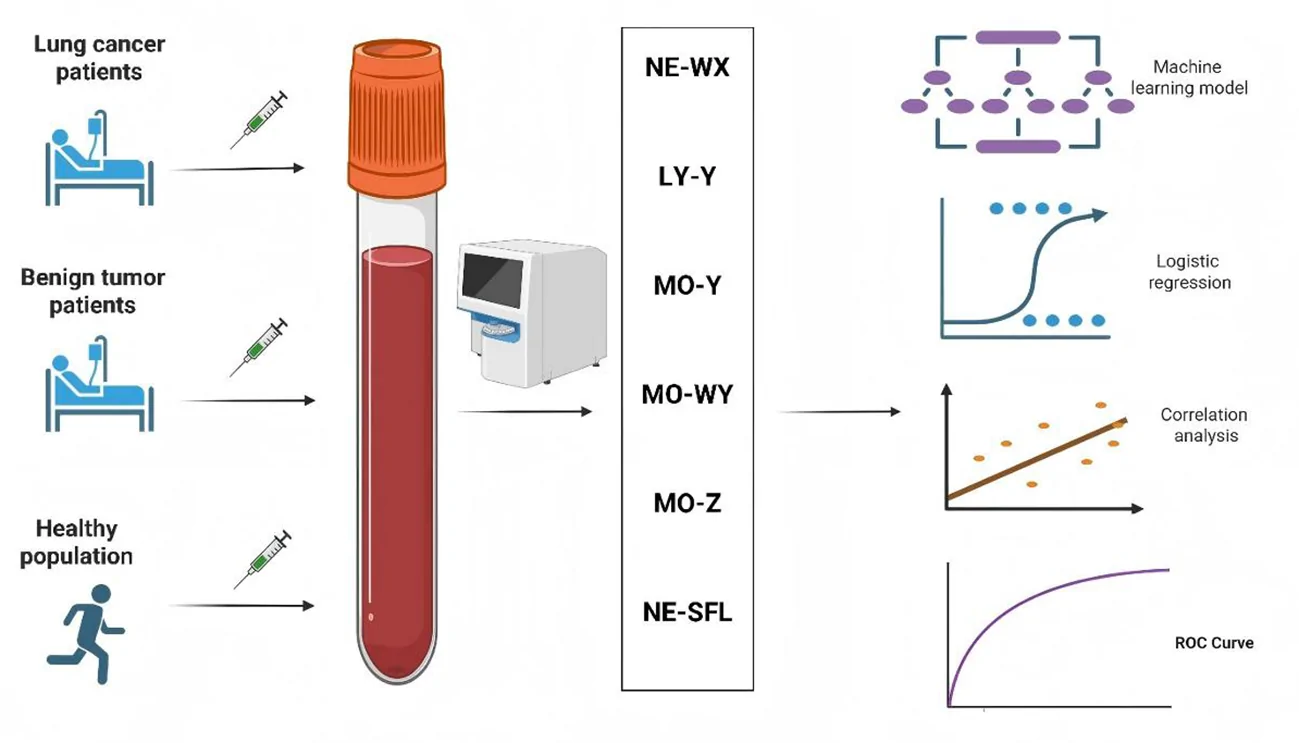
Flow chart of the study.

A machine-learning model was established. The available data were randomly divided into the training and testing sets following a 7:3 ratio using the hold-out method. Data from the training set (n=538) were used for model fitting, whereas those from the testing set (n=231) for model validation. Nine algorithms were used: support vector machine (SVM), gradient boosting machine (GBM), artificial neural network (ANN), random forest (RF), extreme gradient boosting (XGBoost), K-nearest neighbor (KNN), adaptive boosting (AdaBoost), light gradient boosting machine (LightGBM) and CatBoost. All hyperparameters were exclusively tuned and finalized in the training data set via cross-validation without any involvement of the independent testing set. Specifically, a 10-fold cross-validation with five repetitions combined with grid search was adopted for model parameter optimization using the caret package. For XGBoost, LightGBM, and CatBoost algorithms, hyperparameter tuning was performed through 10-fold cross-validation with early stopping strategy to prevent overfitting. Additionally, all six predictive features (NE-WX, LY-Y, MO-WY, MO-Y, MO-Z, and NE-SFL) were pre-specified based on multivariable logistic regression analysis and fully incorporated into the models. The event-per-variable ratio in the training set was approximately 36, ensuring robust model training and stable predictive efficacy. To evaluate the diagnostic performance of the model, AUC, accuracy, sensitivity, specificity, F1-score and precision were calculated. Statistical comparisons of AUCs derived from nine machine learning models were conducted using the DeLong test. Decision curve analysis (DCA) was applied to quantify clinical utility and net benefit. Calibration curves were plotted to assess the consistency between model-predicted probabilities and observed event probabilities, thereby verifying the predictive reliability of the established models. The Shapley additive explanation (SHAP) framework was applied to interpret the diagnostic values contributed by each of the six leukocyte parameters in identifying LC. The study flowchart is outlined in **Figure 1**.

## 3 Results

### 3.1 Comparisons of basic demography among the LC, benign lung tumor, and healthy control groups

**Figure 2:**
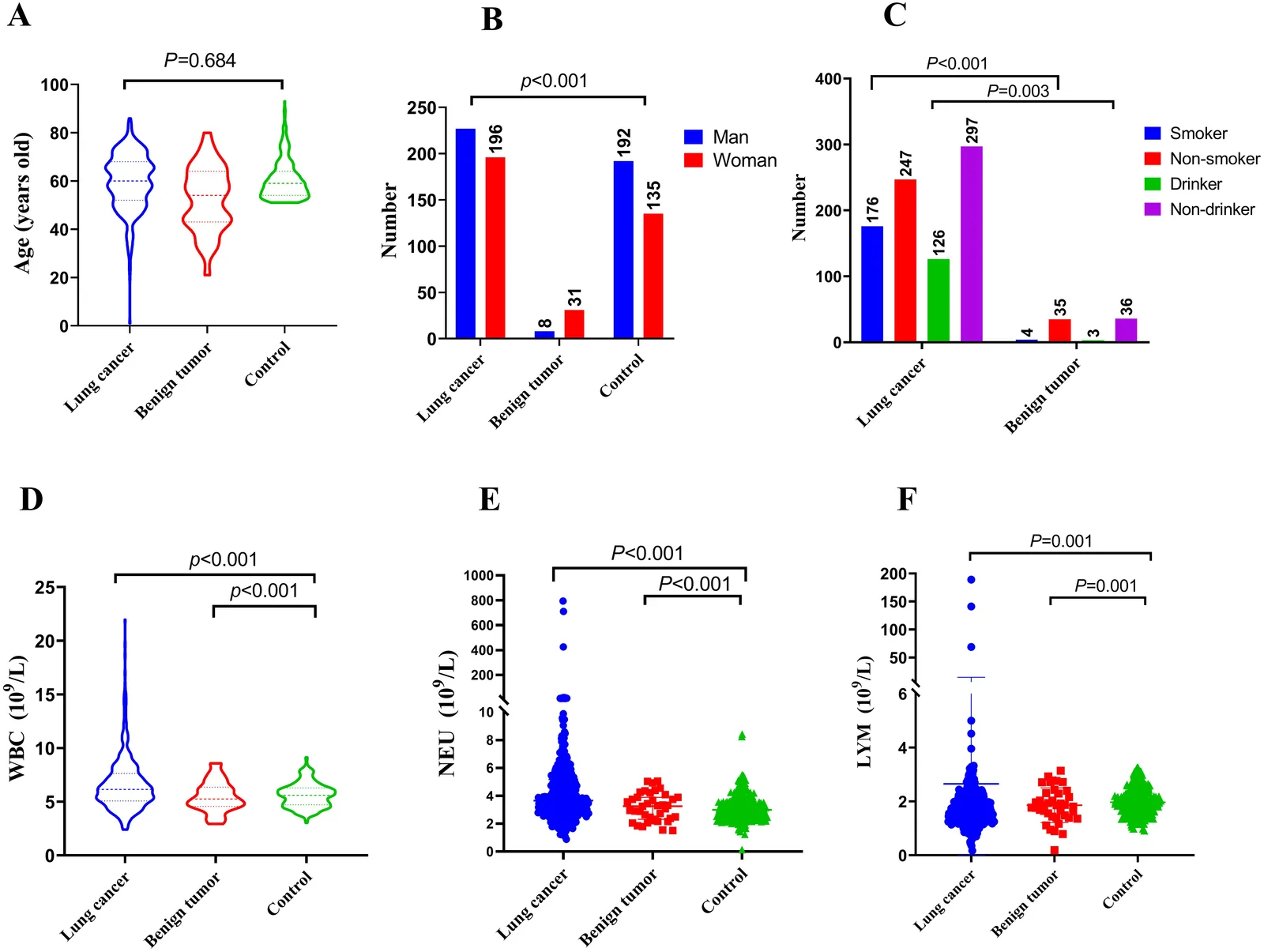
Comparisons of basic demography among the three groups. **(A)** Age; **(B)** Sex; **(C)** Smoker and drinker; **(D)** WBC; **(E)** NEU; **(F)** LYM.

**Figure 3:**
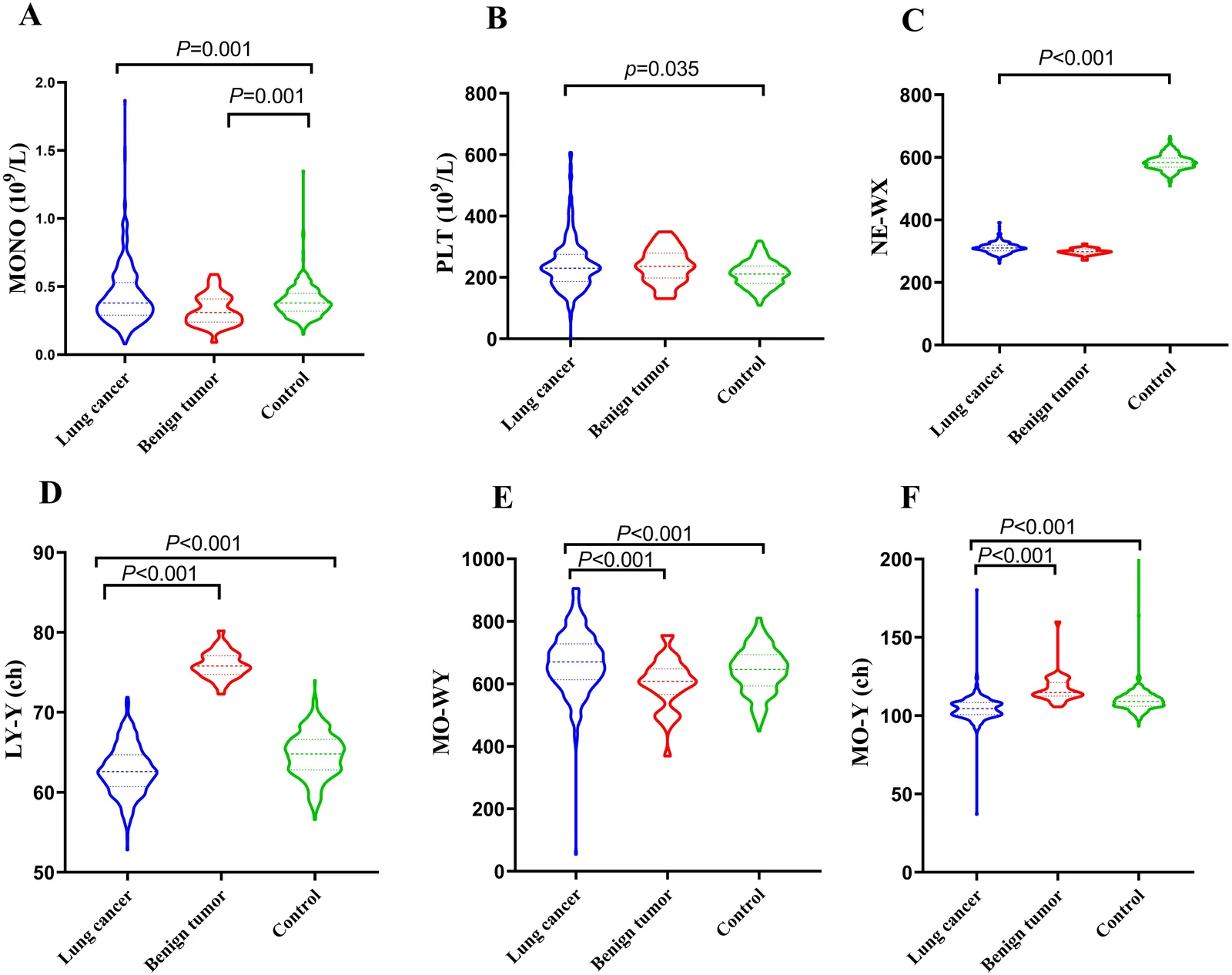
Comparisons of laboratory parameters among the three groups. **(A)** MONO; **(B)** PLT; **(C)** NE-WX; **(D)** LY-Y; **(E)** MO-WY; **(F)** MO-Y.

**Figure 4:**
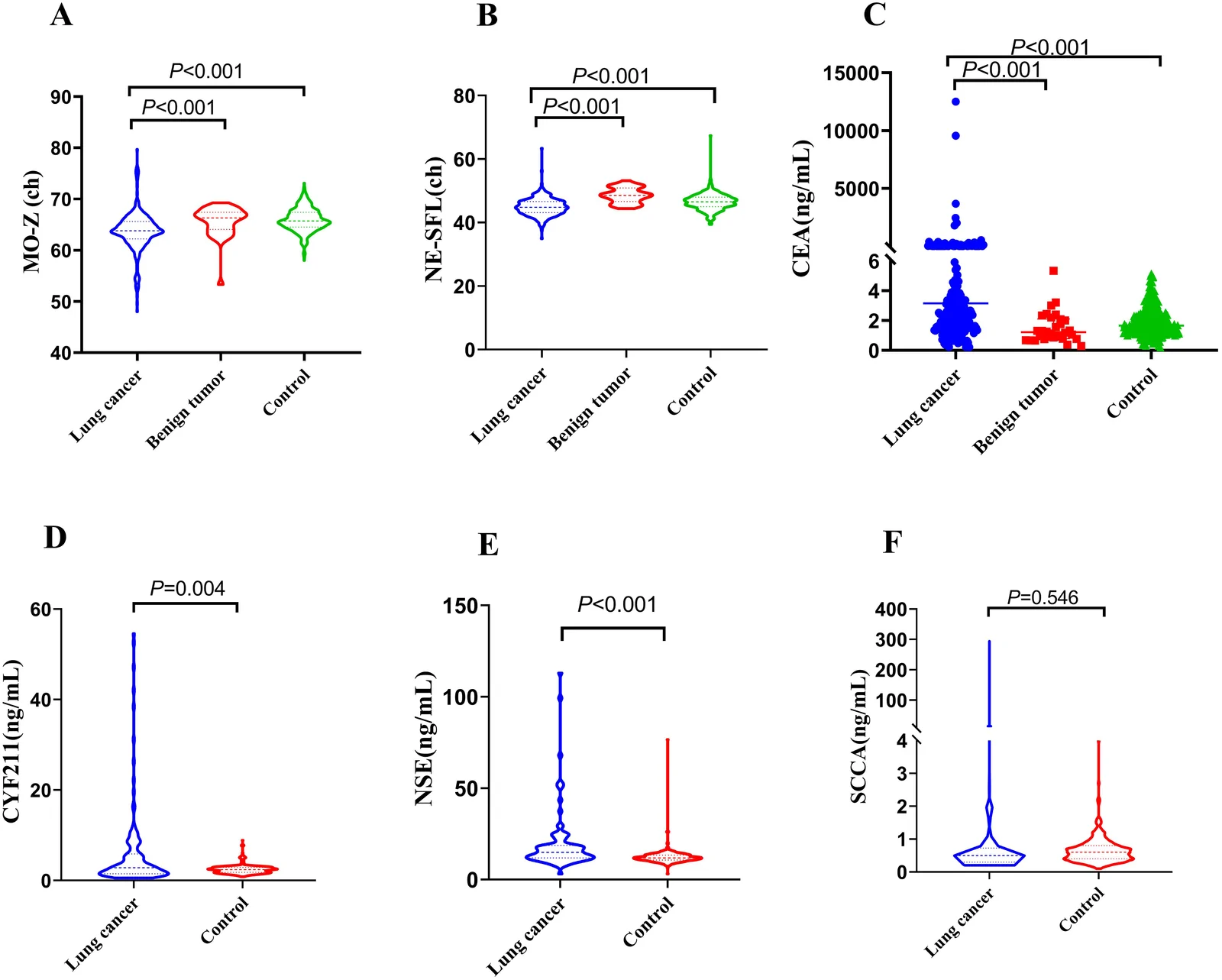
Comparisons on laboratory parameters among the three groups (continued). **(A)** MO-Z; **(B)** NE-SFL; **(C)** CEA; **(D)** CYF211; **(E)** NSE; **(F)** SCCA.

**Figure 5:**
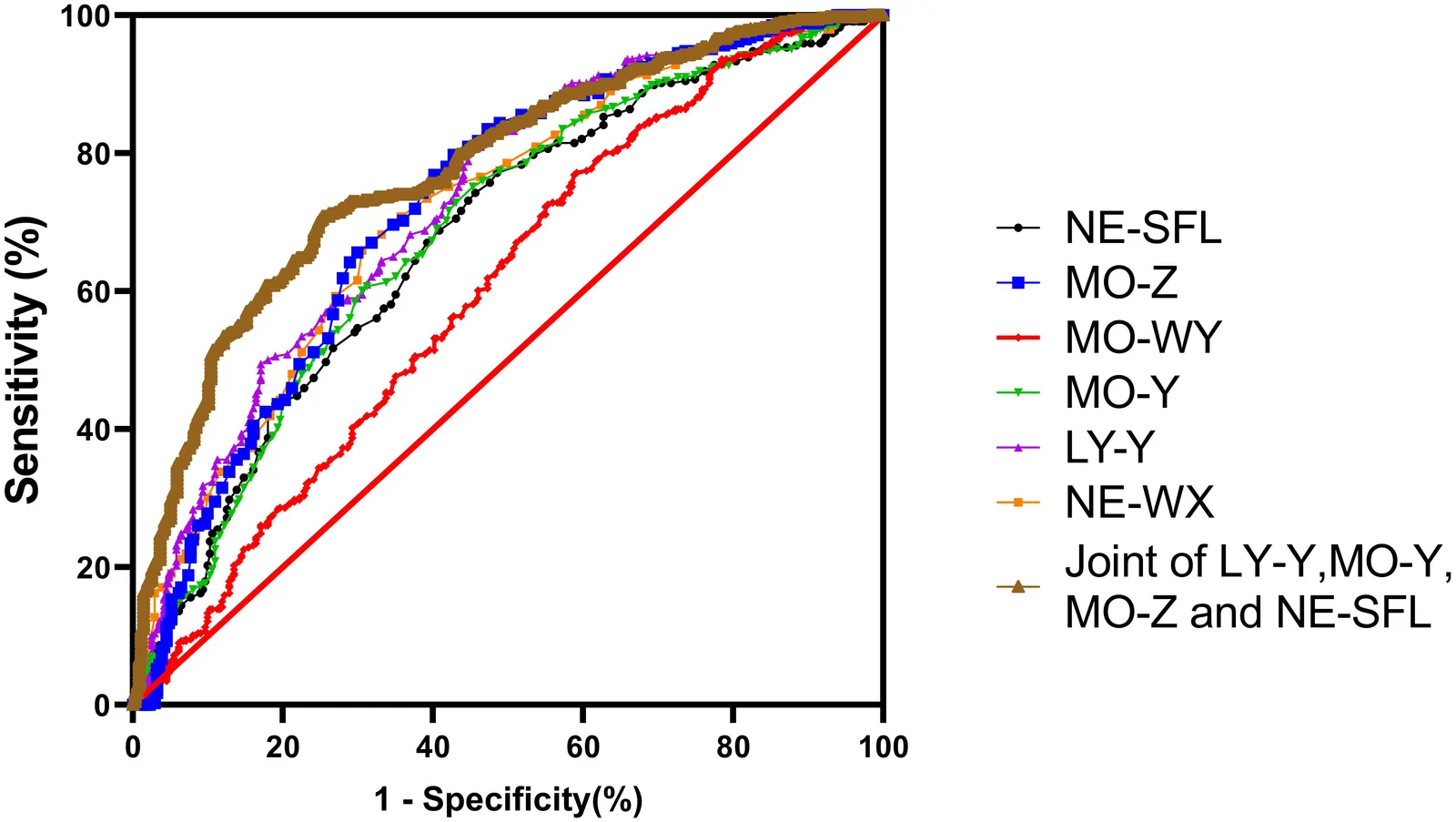
Receiver operating characteristic (ROC) curve of joint and single diagnostic for lung cancer.

Compared to the healthy control group, significant differences were observed in smoking history, drinking history, NEU, LYM, PLT, NE-WX, LY-Y, MO-WY, MO-Y, MO-Z, NE-SFL, CEA, CYF211, and NSE levels in the LC group (*p* < 0.05). WBC, NEU, PLT, NE-WX, MO-WY, CEA, CYF211, and NSE levels in the LC group were higher than those in the control group, whereas the levels of LYM, LY-Y, MO-Y, MO-Z, and NE-SFL were lower than those in the control group (*p* < 0.05). When compared with the healthy control group, significant differences were observed in MONO, LY-Y, MO-Y, and NE-SFL in the benign lung tumor group (*p* < 0.05). The levels of LY-Y, MO-Y, and NE-SFL were significantly higher in patients with benign lung tumors than in healthy controls, whereas the MONO levels were significantly reduced (*p* < 0.05). Compared with the benign lung tumor group, significant differences were observed in smoking history, drinking history, WBC, NEU, MONO, NE-WX, LY-Y, MO-WY, MO-Y, MO-Z, NE-SFL, and CEA levels in the LC group (*p* < 0.05). Smoking history, drinking history, as well as the WBC, NEU, MONO, NE-WX, MO-WY, and CEA levels in the LC group were higher than those in the benign lung tumor group, whereas the LY-Y, MO-Y, MO-Z, and NE-SFL levels were lower (*p* < 0.05). Details are shown in **Figures 2**–**5** and **Appendix Table 1**.

### 3.2 Results of the peripheral blood leukocyte parameters and diagnosis of LC

Multivariable analysis, controlling for cytokine parameters, including WBC, NEU, and LYM, identified NE-WX, LY-Y, MO-WY, MO-Y, MO-Z, and NE-SFL as being associated with LC, with NE-SFL and MO-Z remaining significant independent factors (*p* = 0.036, odds ratio [OR]: 0.912; *p* < 0.001, OR: 0.793, respectively) (**Appendix Table 2**).

### 3.3 ROC curve analysis of the peripheral blood leukocyte parameters for diagnosing LC

When detected alone, MO-Y showed the best diagnostic performance, with an AUC of 0.787 (95% confidence interval [CI]: 0.697–0.974), specificity of 82.0%, and a Youden index of 0.371. MO-Z had an AUC of 0.726 (95% CI: 0.686–0.765), a specificity of 80.4%, and a Youden index of 0.375. The combination panel of LY-Y, MO-Y, MO-Z, and NE-SFL achieved an AUC of 0.897 in the diagnosis of LC (95% CI: 0.773–0.941), with a sensitivity of 70.6%, specificity of 78.9%, and a Youden index of 0.495 (**Figure 5**; **Appendix Table 3**).

### 3.4 Results from machine-learning models

#### 3.4.1 Discriminative performance

The performance of nine machine-learning models was evaluated on both the training and testing sets, with detailed metrics summarized in **Appendix Tables 4, 5**.

**Figure 6:**
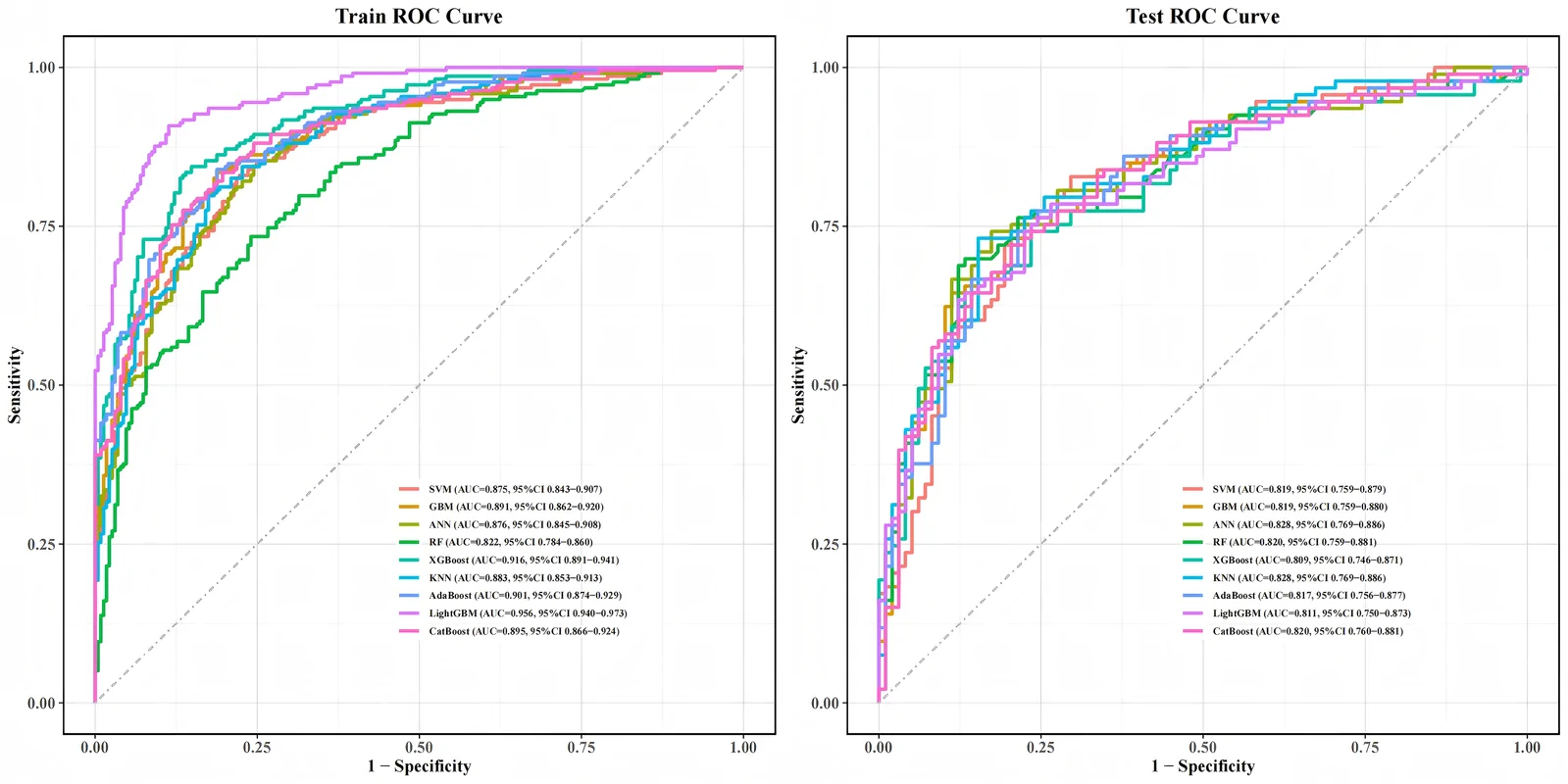
Comparison of receiver operating characteristic (ROC) curves between the training and testing sets.

All nine machine learning models achieved favourable discriminative capacity on the independent testing set, with AUC values ranging from 0.809 (XGBoost) to 0.828 (ANN/KNN). Pairwise comparisons via the DeLong test failed to detect significant inter-model differences, demonstrating that the predictive strategy was robust and did not rely on any algorithm (*p*>0.05). Sensitivity analysis using repeated dataset splitting with five different random seeds further verified the robustness of the findings. The mean AUC of all models was 0.847–0.860, with standard deviations limited to 0.027–0.039, demonstrating high stability of model performance across different data partitioning strategies (**Figure 6**; **Appendix Tables 6, 7**).

#### 3.4.2 Calibration and reliability

**Figure 7:**
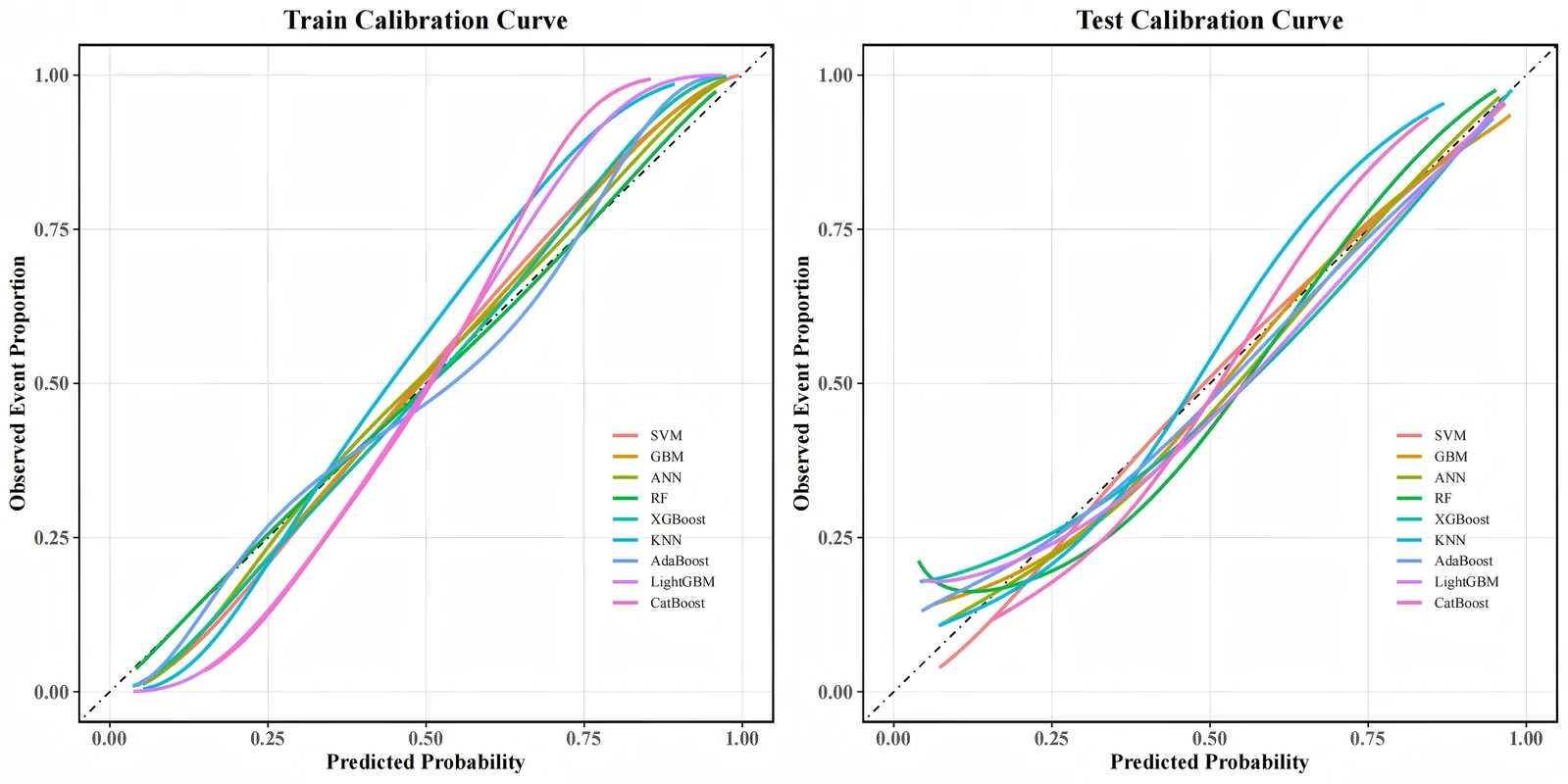
Calibration between the training and testing sets.

**Figure 8:**
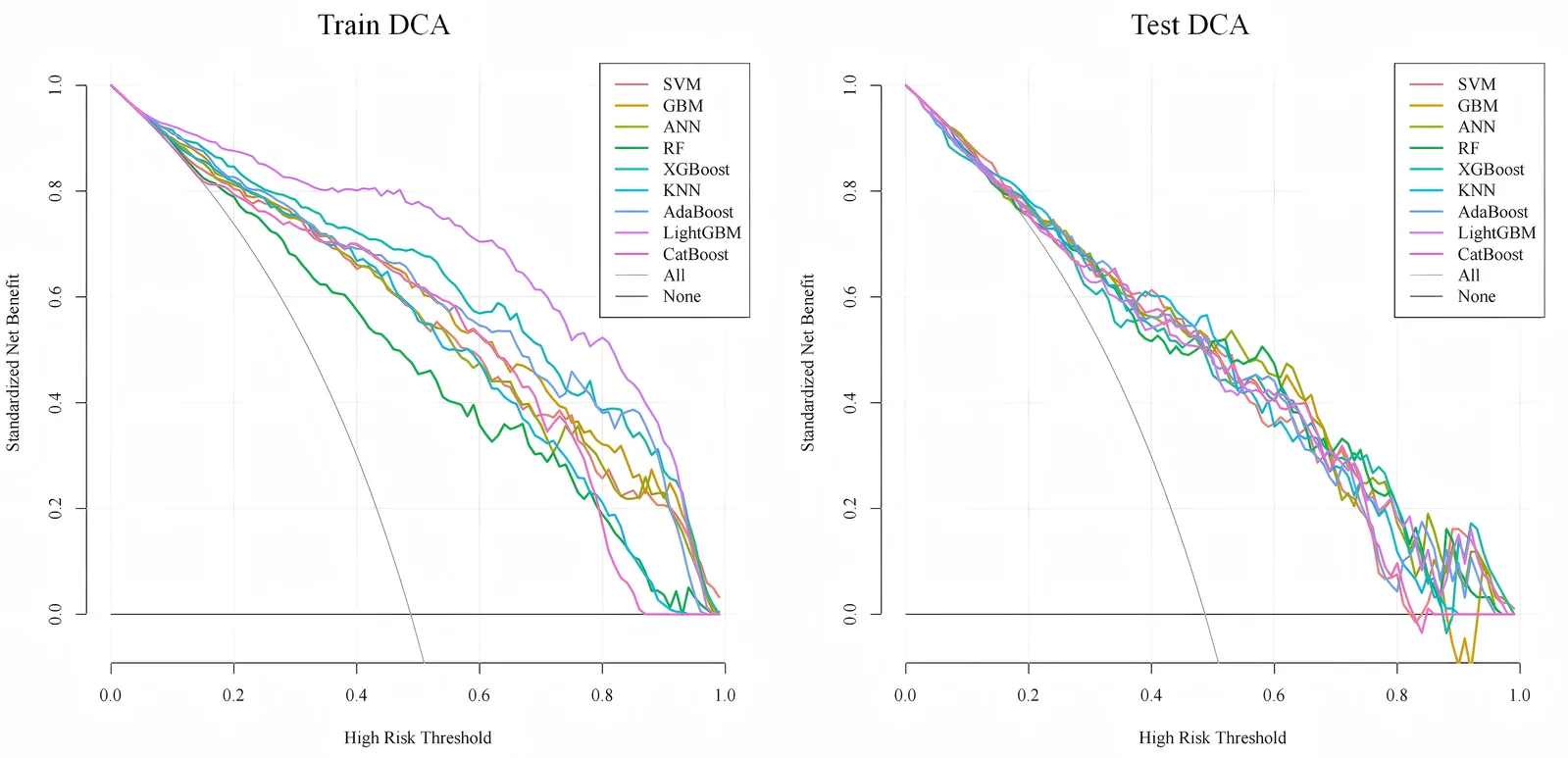
Decision curve analysis (DCA) between the training and testing sets.

The RF, SVM, ANN, and GBM models yielded satisfactory calibration, with calibration slopes approximating the ideal value of 1. Notably, the RF model displayed strong concordance between the out-of-bag (OOB) AUC in the training set (0.822) and the testing set AUC (0.820), verifying authentic and reliable performance estimation without obvious overfitting (**Figures 7**, **8**).

#### 3.4.3 Variable importance

**Figure 9:**
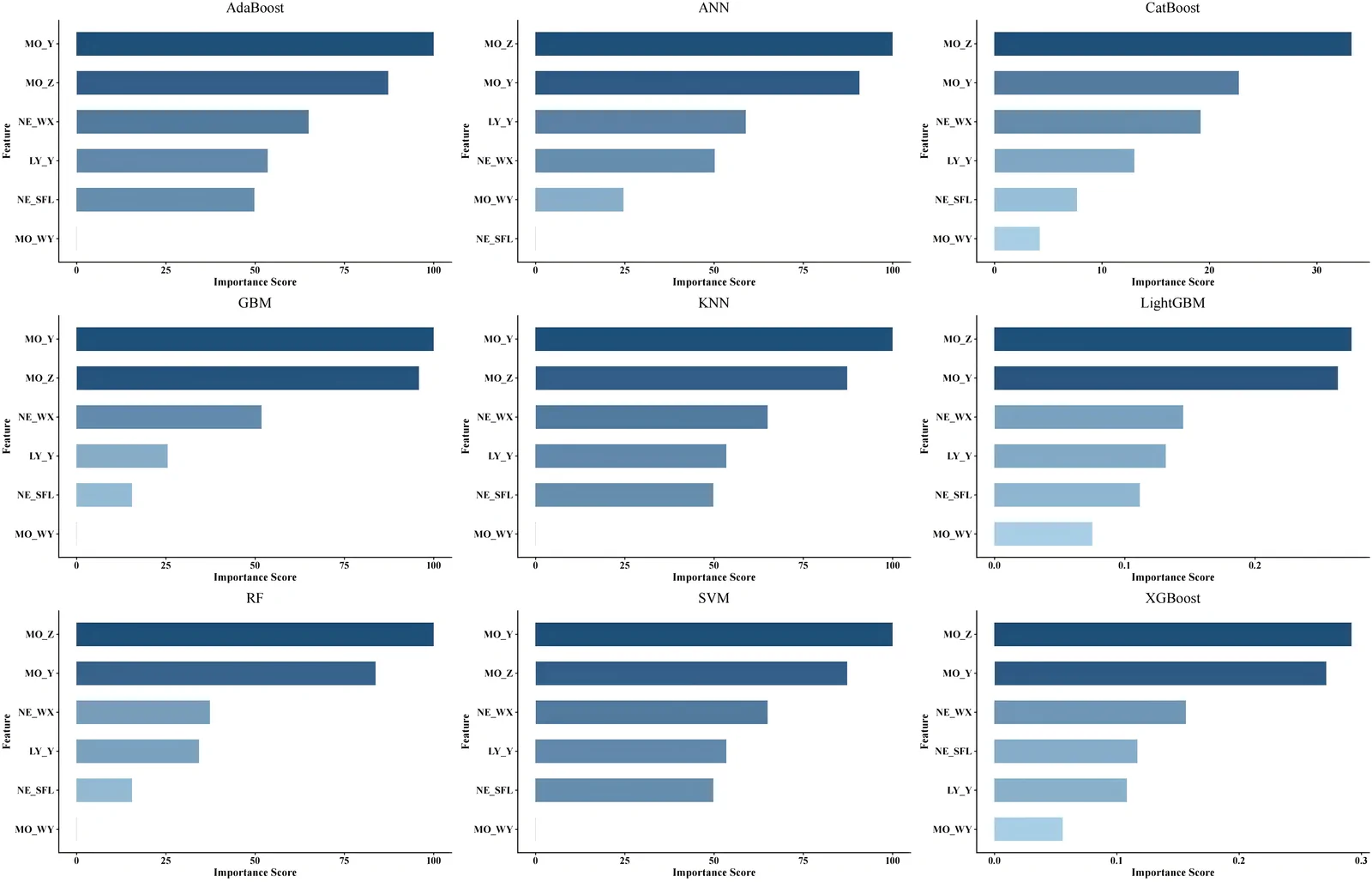
Bar chart showing the relative importance ranking of variables in the models.

**Figure 10:**
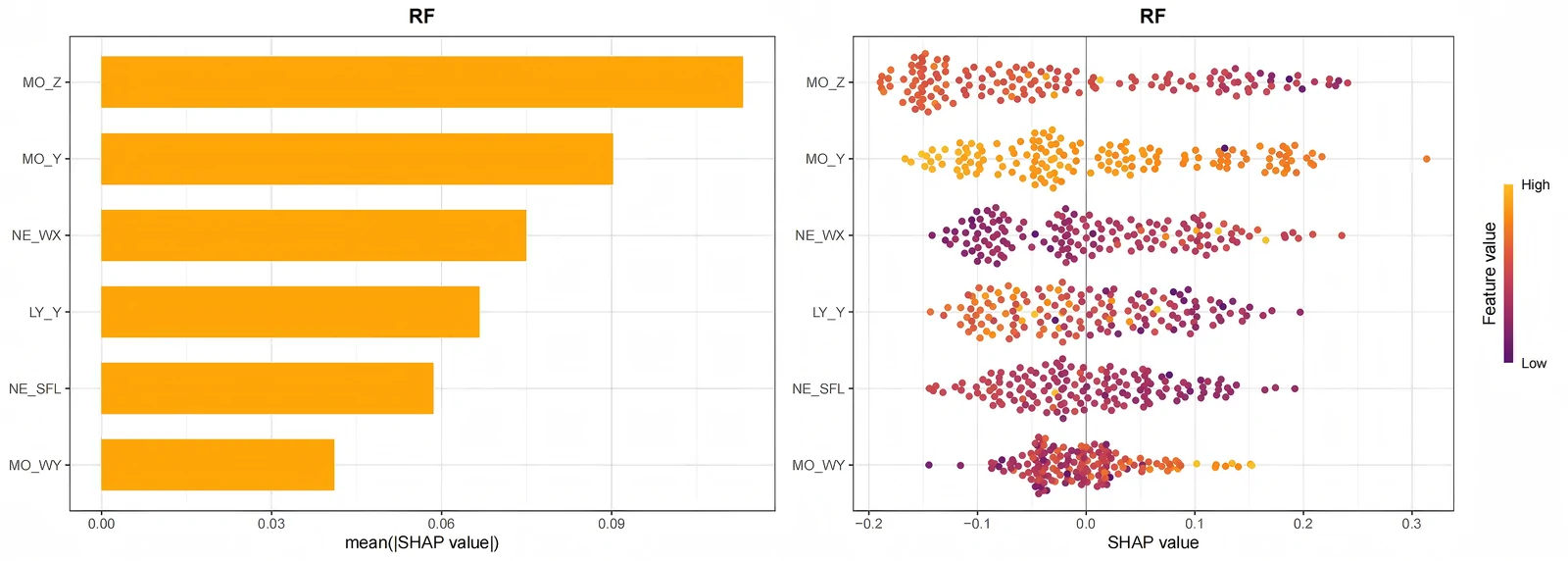
Results of Shapley additive explanation (SHAP) analysis of the random forest (RF) model.

Feature importance bar charts summarized the predictive contribution of six leukocyte indicators across all nine machine learning classifiers. Across nearly all algorithms, MO-Z and MO-Y ranked as the top two most influential predictors. NE-WX was consistently the third important feature across models. This cross-model consistency confirmed that monocyte morphological parameters (MO-Z, MO-Y) are the top predictive variables. SHAP analysis derived from the RF algorithm was consistent with cross-model variable importance analysis. Both analyses identified MO-Z, MO-Y, and NE-WX as the top predictive predictors, confirming the stable predictive contribution of these routine blood routine parameters. This finding lays a solid foundation for predictive modeling built on conventional hematological cell indicators (**Figures 9**, **10**).

## 4 Discussion

The CPD encompasses a series of research parameters provided by the Sysmex X Series fully automatic blood analyzer. Routine testing on this platform automatically yields CPD parameters, helping avoid repetitive blood tests and additional costs. These structural parameters are closely related to cell size, complexity, and nucleic acid content, and therefore, can reflect specific and individual morphological changes in the WBC count (15). Accumulating evidence underscores the critical involvement of inflammatory responses in the pathogenesis and progression of LC. As a core component of the inflammatory response, WBCs show quantitative and functional changes closely related to the pathological process of LC. In advanced NSCLC, a high neutrophil-to-white blood cell ratio, high neutrophil-to-lymphocyte ratio, high monocyte-to-lymphocyte ratio, and low lymphocyte-to-white blood cell ratio served as independent factors for an unfavorable overall survival outcome (16). According to the UK Biobank data, elevated WBC counts may increase the risk of LC (17). The association between WBC count and LC and its pathological classification through Mendelian randomization analysis suggests that increased WBC and monocyte counts are associated with an elevated risk of lung adenocarcinoma (18). Our results are closely aligned with prior reports that noted decreased lymphocyte and elevated neutrophil counts in advanced LC (19). The WBC structural parameter CPD undergoes dynamic alterations throughout the development and progression of LC. In this study, NE-WX expression increased in the LC group compared with that in the control group, and the difference was significant, indicating increased complexity within neutrophils. MO-WY significantly increased, reflecting a broader fluorescence distribution width in the monocyte region, indicative of immature monocyte development. A significant decrease in LY-Y was observed in the LC group compared with controls, which may be related to the tumor immune escape mechanism. Compared to the control group, MO-Y and MO-Z in the LC group decreased significantly, which may be related to the phenotypic polarization and dysfunction of monocytes in patients with LC. Moreover, compared to the control group, NE-SFL in the LC group significantly decreased, indicating altered chromatin, DNA, and RNA concentrations in neutrophils, which may be related to immune imbalance and immunosuppression in patients with LC.

In a systematic review, Xu et al. demonstrated that solid tumors secrete a range of cytokines, including interleukin-6, C-C motif chemokine ligand 2, and tumor growth factor beta (TGF)-β, which continuously modulate the activation, maturation, and polarization of circulating neutrophils, monocytes, and lymphocytes, altering the micromorphological characteristics of peripheral immune cells, such as granularity, nucleic acid content, and cell size (20). The infiltration burden of macrophages, activated neutrophils, and exhausted T cells within tumor lesions is closely associated with dynamic changes in peripheral CPD parameters (NE-WX, MO-Z, MO-Y, and LY-Y). Accordingly, peripheral blood cell morphological parameters may indirectly reflect the severity of the immunosuppressive tumor microenvironment (TME). These findings provide robust theoretical support for the differential alterations in MO-Z and NE-WX observed in our study. Notably, the core mechanisms underlying TME remodeling are common across solid tumors and are therefore applicable to the initiation and progression of LC.

In recent years, machine learning has been extensively adopted for early diagnosis and risk stratification of LC. Prior studies have constructed computer-aided diagnostic systems based on RF algorithms to differentiate between benign and malignant pulmonary nodules, reaching a sensitivity of 92.4% and specificity of 94.8% (21–24). As systematically summarized by Liu et al., integrated algorithms including GBM, XGBoost, SVM and KNN exhibit unique advantages in excavating high-dimensional tumor biomarkers, whereas single molecular indicators often yield unsatisfactory predictive efficacy; multi-marker signatures screened by artificial intelligence enable more accurate patient risk stratification (25). Following this theoretical paradigm, we compared nine machine-learning algorithms by inputting a panel of peripheral blood CPD immune parameters (MO-Z, NE-WX, MO-Y, LY-Y) and ultimately identified RF as the optimal predictive model for LC. To mitigate the inherent overfitting risk of tree-based algorithms highlighted in Liu’s review, stratified 10-fold cross-validation was strictly performed for internal validation.

From the perspective of model interpretability, we adopted SHAP-based variable contribution analysis to quantify the independent predictive weight of each CPD feature, complying with the consensus that explainable artificial intelligence is essential for clinical translational research. Both global feature importance ranking and SHAP analysis consistently confirmed MO-Y and MO-Z as the leading predictive biomarkers of LC. Mechanistically, this finding can be well interpreted by the regulatory axis reported by Li et al. (26). Lung adenocarcinoma stem cells remodel the intratumoral immune micro-environment and stimulate massive secretion of inflammatory cytokines, which drives phenotypic reprogramming of circulating monocytes, macrophages and neutrophils. This tumor-originated systemic inflammatory cascade disrupts normal monocyte maturation and leads to abnormal alterations in MO-Z, alongside with significant fluctuations in other CPD markers (MO-WY, MO-Y, NE-WX). Collectively, these peripheral blood morphological parameters act as non-invasive readouts that indirectly mirror the severity of intratumoral immunosuppression and stem cell activity.

Some limitations of this study should be considered when interpreting the findings. First, the sample size of patients with benign lung tumors was relatively small. In clinical practice, patients with suspected benign lung lesions are generally managed with conservative surveillance or interventional treatment rather than surgical resection, and histopathological confirmation of benign lung tumors is available only for those who undergo surgical resection. Second, because this was a retrospective observational study, complete matched data on C-reactive protein levels, procalcitonin levels, and erythrocyte sedimentation rate were unavailable for all participants, precluding quantitative evaluation of subclinical inflammatory interference and potentially introducing selection bias. Third, all patient data were retrospectively collected from a single medical center, and no independent external multicenter cohort was included for out-of-sample validation. Future prospective multicenter cohort studies are needed to externally validate the established LC risk prediction model and further evaluate its clinical utility.

## 5 Conclusion

In conclusion, the WBC parameter group reflects not only the body’s inflammatory state, but also its close association with the tumor micro-environment and immune escape. It has good predictive value for diagnosing LC and holds promise for improving the diagnosis of LC in clinical practice. Our study demonstrates that the RF model serves as a promising machine learning algorithm for lung cancer diagnosis.

## Data Availability

The original contributions presented in the study are included in the article/**Supplementary Material**. Further inquiries can be directed to the corresponding author.
