# Supplementary material for: Diagnostic value of peripheral blood leukocyte parameters, NE-WX, LY-Y, MO-WY, MO-Y, MO-Z, and NE-SFL, in lung cancer
Source: Front Oncol. 2026 Aug 26;16:1793608. doi: 10.3389/fonc.2026.1793608 (PMC13553461; doi:10.3389/fonc.2026.1793608)
Supplement: Supplementary file 1 [file Table1.docx]

**Appendix Table 1.** Comparison of clinical demographics among the lung cancer, benign, and healthy control groups

| Variables | Lung cancer group  n=423 | Lung benign group  n=39 | Control group  n=346 | *H/F/χ2* | *p­-*value |
| --- | --- | --- | --- | --- | --- |
| Age (years) (M25, M75) | 60 (52, 68) | 54 (43, 64) | 59 (54, 64) | 11.97. | 0.224 |
| Men [Number (%)] | 227 (53.66) | 8 (20.51) | 201 (58.1) | 1.512 | 0.219 |
| Smoker [Number (%)] | 176 (41.61)^b^ | 4 (10.26) | - | 14.8 | <0.001 |
| Drink [Number (%)] | 126 (29. 79)^b^ | 3 (7.69) | - | 8.7 | 0.003 |
| WBC  (*10^9^/L) | 6.25 (4.98,  7.49) ^ab^ | 5.48 (4.77, 7.04) | 5.61 (4.72, 6.28) | 35.7 | <0.001 |
| NEU  (*10^9^/L) | 3.54 (2.89, 5.16)^ab^ | 3.30  (2.43, 4.15) | 2.99  (2.47, 3.61) | 71.6 | <0.001 |
| LYM  (*10^9^/L) | 1.65 (1.30, 2.06)^a^ | 1.92  (1.46, 2.72) | 1.95  (1.62, 2.29) | 42.8 | <0.001 |
| MONO  (*10^9^/L) | 0.40  (0.31, 0.55)^b^ | 0.31  (0.22, 0.42)^a^ | 0.38  (0.32, 0.45) | 13.3 | 0.001 |
| PLT (*10^9^/L) | 233 (192, 270)^a^ | 245 (212, 278) | 211  (181, 237) | 27.2 | <0.001 |
| NE-WX | 309 (301, 317) ^ab^ | 298.0 (294, 308) | 299 (290, 307) | 96.2 | <0.001 |
| LY-Y | 62.7 (60.9, 64.9) ^ab^ | 69.4 (65.9, 70.9) ^a^ | 64.8 (62.8, 66.6) | 110.9 | <0.001 |
| MO-WY | 666 (602, 727) ^ab^ | 613 (589, 654) | 646 (593, 693) | 29.9 | <0.001 |
| MO-Y | 104.2  (100.7, 108.7) ^ab^ | 114.6  (112.6, 120.7) ^a^ | 109.1  (106.0, 112.6) | 155.2 | <0.001 |
| MO-Z | 63.7 (62.2, 65.3) ^ab^ | 66.4 (64.5, 67.8) | 65.7 (64.5, 67.4) | 100.6 | <0.001 |
| NE-SFL | 44.6 (42.9, 46.6) ^ab^ | 48.8 (47.3, 50.8) ^a^ | 46.5 (45.0, 48.0) | 88.0 | <0.001 |
| CEA | 2.99 (1.55, 13.12) ^ab^ | 1.25 (0.78, 2.05) | 1.66 (1.13, 2.33) | 81.8 | <0.001 |
| CYF211 | 2.83 (1.48, 5.88) ^a^ | - | 2.41 (1.8, 2.89) | 15.4 | <0.001 |
| NSE | 14.98  (11.90, 18.70) ^a^ | - | 11.91  (10.73, 13.37) | 70.9 | <0.001 |
| SCCA | 0.50 (0.30, 0.73) | - | 0.60 (0.40, 0.80) | 1.359 | 0.244 |

Note：^a^ *p*<0.05, case vs control; ^b^ *p<*0.05 case vs benign

**Appendix Table 2.** Results of multivariable analysis for biomarkers of lung cancer diagnosis

| Items | *β* | *SE* | *p*-value | OR | 95% CI |
| --- | --- | --- | --- | --- | --- |
| NE-WX | 0.045 | 0.007 | <0.001 | 1.046 | 1.031–1.062 |
| LY-Y | -0.168 | 0.037 | <0.001 | 0.845 | 0.786–0.910 |
| MO-WY | 0.003 | 0.001 | 0.018 | 1.003 | 1.001–1.005 |
| MO-Y | -0.065 | 0.020 | 0.001 | 0.937 | 0.90–0.975 |
| MO-Z | -0.232 | 0.036 | <0.001 | 0.793 | 0.739–0.850 |
| NE-SFL | -0.092 | 0.044 | 0.036 | 0.912 | 0.837–0.994 |

**Appendix Table 3.** Results of ROC curve analyses for the six parameters to diagnose lung cancer

| Parameters | Cut-off | AUC | 95% CI | *p*-value | Sensitivity | Specificity | Youden index |
| --- | --- | --- | --- | --- | --- | --- | --- |
| NE-WX | 305.5 | 0.717 | 0.677–0.756 | <0.001 | 0.643 | 0.716 | 0.359 |
| MO-WY | 693.5 | 0.602 | 0.551–0.639 | <0.001 | 0.412 | 0.761 | 0.173 |
| LY-Y | 64.05 | 0.682 | 0.641–0.724 | <0.001 | 0.694 | 0.602 | 0.296 |
| MO-Y | 105.25 | 0.787 | 0.697–0.974 | <0.001 | 0.552 | 0.820 | 0.371 |
| MO-Z | 64.25 | 0.726 | 0.686–0.765 | <0.001 | 0.571 | 0.804 | 0.375 |
| NE-SFL | 45.05 | 0.678 | 0.635–0.718 | <0.001 | 0.545 | 0.743 | 0.288 |
| LY-Y+MO-Y+MO-Z+NE-SFL | | 0.897 | 0.773–0.941 | <0.001 | 0.706 | 0.789 | 0.495 |

**Appendix Table 4.** Performance of the nine models on the training set

| **Model** | **AUC** | **Sensitivity** | **Specificity** | **Accuracy** | **Precision** | **F1-score** |
| --- | --- | --- | --- | --- | --- | --- |
| SVM | 0.875 | 0.830 | 0.777 | 0.803 | 0.780 | 0.804 |
| GBM | 0.891 | 0.835 | 0.812 | 0.823 | 0.809 | 0.822 |
| ANN | 0.876 | 0.844 | 0.755 | 0.799 | 0.767 | 0.803 |
| RF | 0.822 | 0.734 | 0.760 | 0.747 | 0.744 | 0.739 |
| XGBoost | 0.916 | 0.844 | 0.852 | 0.848 | 0.844 | 0.844 |
| KNN | 0.883 | 0.798 | 0.825 | 0.812 | 0.813 | 0.806 |
| AdaBoost | 0.901 | 0.839 | 0.812 | 0.826 | 0.810 | 0.824 |
| LightGBM | 0.956 | 0.908 | 0.886 | 0.897 | 0.884 | 0.896 |
| CatBoost | 0.895 | 0.775 | 0.865 | 0.821 | 0.845 | 0.809 |

SVM, Support Vector Machine; GBM, Gradient Boosting Machine; ANN, Artificial Neural Network; RF, Random Forest; XGBoost, Extreme Gradient Boosting; KNN, K-nearest neighbour; Adaboost, Adaptive Boosting; LightGBM, Light Gradient Boosting Machine; AUC, area under the curve.

**Appendix Table 5.** Performance of the nine models on the testing set

| **Model** | **AUC** | **Sensitivity** | **Specificity** | **Accuracy** | **Precision** | **F1-score** |
| --- | --- | --- | --- | --- | --- | --- |
| SVM | 0.819 | 0.806 | 0.714 | 0.759 | 0.728 | 0.765 |
| GBM | 0.819 | 0.753 | 0.755 | 0.754 | 0.745 | 0.749 |
| ANN | 0.828 | 0.806 | 0.694 | 0.749 | 0.714 | 0.758 |
| RF | 0.820 | 0.763 | 0.786 | 0.775 | 0.772 | 0.768 |
| XGBoost | 0.809 | 0.720 | 0.765 | 0.743 | 0.744 | 0.732 |
| KNN | 0.828 | 0.774 | 0.755 | 0.764 | 0.750 | 0.762 |
| AdaBoost | 0.817 | 0.763 | 0.755 | 0.759 | 0.747 | 0.755 |
| LightGBM | 0.811 | 0.742 | 0.765 | 0.754 | 0.750 | 0.746 |
| CatBoost | 0.820 | 0.688 | 0.806 | 0.749 | 0.771 | 0.727 |

**Appendix Table 6.** Pairwise DeLong comparisons of the nine models on the testing set

| **Model A** | **Model B** | **AUC A** | **AUC B** | **Diff** | ***P*** |
| --- | --- | --- | --- | --- | --- |
| GBM | XGBoost | 0.819 | 0.809 | 0.011 | 0.1535 |
| XGBoost | CatBoost | 0.809 | 0.82 | -0.012 | 0.1823 |
| ANN | XGBoost | 0.828 | 0.809 | 0.019 | 0.203 |
| XGBoost | KNN | 0.809 | 0.828 | -0.019 | 0.2253 |
| ANN | LightGBM | 0.828 | 0.811 | 0.016 | 0.2583 |
| KNN | LightGBM | 0.828 | 0.811 | 0.016 | 0.2622 |
| RF | XGBoost | 0.82 | 0.809 | 0.011 | 0.3118 |
| RF | LightGBM | 0.82 | 0.811 | 0.009 | 0.3136 |
| ANN | AdaBoost | 0.828 | 0.817 | 0.011 | 0.3164 |
| XGBoost | AdaBoost | 0.809 | 0.817 | -0.008 | 0.3235 |
| LightGBM | CatBoost | 0.811 | 0.82 | -0.009 | 0.3753 |
| KNN | AdaBoost | 0.828 | 0.817 | 0.011 | 0.3812 |
| GBM | ANN | 0.819 | 0.828 | -0.008 | 0.4238 |
| SVM | ANN | 0.819 | 0.828 | -0.008 | 0.4313 |
| GBM | LightGBM | 0.819 | 0.811 | 0.008 | 0.4399 |
| GBM | KNN | 0.819 | 0.828 | -0.009 | 0.4912 |
| RF | KNN | 0.82 | 0.828 | -0.008 | 0.5128 |
| ANN | CatBoost | 0.828 | 0.82 | 0.007 | 0.5471 |
| KNN | CatBoost | 0.828 | 0.82 | 0.008 | 0.5486 |
| SVM | KNN | 0.819 | 0.828 | -0.009 | 0.5509 |
| SVM | XGBoost | 0.819 | 0.809 | 0.011 | 0.5675 |
| ANN | RF | 0.828 | 0.82 | 0.008 | 0.568 |
| AdaBoost | LightGBM | 0.817 | 0.811 | 0.005 | 0.5794 |
| AdaBoost | CatBoost | 0.817 | 0.82 | -0.004 | 0.5831 |
| GBM | AdaBoost | 0.819 | 0.817 | 0.003 | 0.6442 |
| SVM | LightGBM | 0.819 | 0.811 | 0.008 | 0.6666 |
| XGBoost | LightGBM | 0.809 | 0.811 | -0.003 | 0.7195 |
| RF | AdaBoost | 0.82 | 0.817 | 0.003 | 0.7514 |
| SVM | AdaBoost | 0.819 | 0.817 | 0.003 | 0.8633 |
| GBM | CatBoost | 0.819 | 0.82 | -0.001 | 0.8715 |
| GBM | RF | 0.819 | 0.82 | -0.001 | 0.9413 |
| SVM | CatBoost | 0.819 | 0.82 | -0.001 | 0.9526 |
| SVM | RF | 0.819 | 0.82 | -0.001 | 0.9696 |
| ANN | KNN | 0.828 | 0.828 | 0 | 0.9747 |
| RF | CatBoost | 0.82 | 0.82 | 0 | 0.9757 |

**Appendix Table 7.** The mean AUCs of the nine models on the testing set

| **Model** | **n_runs** | **AUC_mean** | **AUC_sd** | **AUC_min** | **AUC_max** |
| --- | --- | --- | --- | --- | --- |
| SVM | 5 | 0.860 | 0.032 | 0.819 | 0.900 |
| AdaBoost | 5 | 0.856 | 0.030 | 0.817 | 0.897 |
| GBM | 5 | 0.856 | 0.029 | 0.819 | 0.89 |
| KNN | 5 | 0.856 | 0.027 | 0.828 | 0.895 |
| XGBoost | 5 | 0.854 | 0.033 | 0.809 | 0.898 |
| CatBoost | 5 | 0.853 | 0.029 | 0.820 | 0.899 |
| ANN | 5 | 0.852 | 0.039 | 0.813 | 0.905 |
| RF | 5 | 0.850 | 0.029 | 0.820 | 0.894 |
| LightGBM | 5 | 0.847 | 0.028 | 0.811 | 0.888 |
